# Different Patterns of Respiration in Rat Lines Selectively Bred for High or Low Anxiety

**DOI:** 10.1371/journal.pone.0064519

**Published:** 2013-05-17

**Authors:** Luca Carnevali, Andrea Sgoifo, Mimosa Trombini, Rainer Landgraf, Inga D. Neumann, Eugene Nalivaiko

**Affiliations:** 1 Department of Neuroscience, University of Parma, Parma, Italy; 2 Max Planck Institute for Psychiatry, Munich, Germany; 3 Department of Behavioural and Molecular Neurobiology, University of Regensburg, Regensburg, Germany; 4 School of Biomedical Sciences and Pharmacy, University of Newcastle, Newcastle, New South Wales, Australia; Max Planck Institute of Psychiatry, Germany

## Abstract

In humans, there is unequivocal evidence of an association between anxiety states and altered respiratory function. Despite this, the link between anxiety and respiration has been poorly evaluated in experimental animals. The primary objective of the present study was to investigate the hypothesis that genetic lines of rats that differ largely in their anxiety level would display matching alterations in respiration. To reach this goal, respiration was recorded in high-anxiety behavior (HAB, n = 10) and low-anxiety behavior (LAB, n = 10) male rats using whole-body plethysmography. In resting state, respiratory rate was higher in HABs (85±2 cycles per minute, cpm) than LABs (67±2 cpm, p<0.05). During initial testing into the plethysmograph and during a restraint test, HAB rats spent less time at high-frequency sniffing compared to LAB rats. In addition, HAB rats did not habituate in terms of respiratory response to repetitive acoustic stressful stimuli. Finally, HAB rats exhibited a larger incidence of sighs during free exploration of the plethysmograph and under stress conditions. We conclude that: i) HAB rats showed respiratory changes (elevated resting respiratory rate, reduced sniffing in novel environment, increased incidence of sighs, and no habituation of the respiratory response to repetitive stimuli) that resemble those observed in anxious and panic patients, and ii) respiratory patterns may represent a promising way for assessing anxiety states in preclinical studies.

## Introduction

In humans, respiratory dysregulation is characteristic of anxiety and related symptoms are a diagnostic feature of several anxiety disorders. Clinical observations of severe respiratory distress in patients with anxiety disorders have stimulated much research to identify respiratory abnormalities associated with this syndrome. Symptoms of hyperventilation [1], breath-to-breath respiratory instability and frequent sighing [2], [3] have commonly been reported in patients with panic disorder, even during panic-free periods [4], [5]. Similar respiratory abnormalities have been found, though less consistently, in patients with generalized anxiety disorders [5]. In addition, exaggerated respiratory arousal in response to psychological stress has been documented in individuals with high trait-anxiety [6].

While a link between respiratory abnormalities and anxiety is well described in humans, preclinical research has just started investigating the respiratory function in animal models of anxiety. Early research into the link between respiration and anxiety in animals was hampered mainly by the lack of techniques that offered sufficient precision with regard to the assessment of standard respiratory indices, relatively easy applicability and non-intrusiveness. Among modern techniques, whole-body plethysmography represents a promising method, as it is entirely non-invasive and thus does not introduce any confounding factor. Using this method, a series of elegant studies conducted by Kinkead and colleagues have recently demonstrated that neonatal maternal separation in rats provokes a respiratory phenotype in adulthood that presents many anxiety-related features. Such animals have altered respiratory responses to hypoxia [7] and hypercapnia [8], with the underlying mechanisms involving both alterations in the chemoreflex circuitry in the lower brainstem [9] and descending influences from the hypothalamus [10]. Further evidence of a link between respiration and anxiety in rats comes from other studies documenting that respiratory parameters (especially the respiratory rate) are strongly affected by conditioned and unconditioned aversive stimuli and by novelty [11], [12].

Given the centrality of breathing in human anxiety and the availability of adequate techniques for the measurement of respiratory indices, research with valid and reliable animal models can offer new important insights into the link between respiration and anxiety.

In this study, we used whole-body plethysmography for conducting a reliable and sensitive analysis of the respiratory function in two Wistar rat lines selectively bred for either high (HAB) or low (LAB) anxiety-related behavior. The HAB/LAB rats have been proved to be extremely divergent in their level of baseline anxiety, as revealed in a variety of behavioral tests (for a review, see [13] and [14]), the differences being robust, consistent, and reliable [15], [16]. Therefore, the use of these psychogenetically selected rats represents, in our view, a valid methodological approach for investigating the respiratory function in animal populations that possess clear differences in their level of baseline anxiety.

Specifically, we tested the hypothesis that in rats different levels of anxiety would be accompanied by matching alterations in respiration. Respiratory function was evaluated in HAB/LAB rats during exploration of the plethysmographic chamber and during exposure to acoustic (predator call), olfactory (cat feces odor) and psychological (restraint) stressful stimuli.

## Methods

### Ethics statement and animals

The experimental protocol described here was approved by the Veterinarian Animal Care and Use Committee of Parma University, and carried out in accordance with the European Community Council Directives of 22 September 2010 (2010/63/UE).

Experiments were carried out on 4-month-old male Wistar rats obtained from the animal facilities of the University of Regensburg (Germany). The animals belonged to two lines selectively bred since 1993 for high or low anxiety-like behavior, as described previously in detail [13], [17], [18]. At their arrival in our laboratory, the HAB (n = 10) and LAB (n = 10) rats used in this study were housed in groups of 3–4 per cage and kept in rooms with controlled temperature (22±2°C) and a reversed light-dark cycle (light on from 19:00 to 7:00 h), with free access to food and water.

### Recordings of respiration and gross motor activity

Respiratory movements were detected using a custom-built whole-body plethysmograph [19]. This consisted of a sealed Perspex cylinder (i.d. 95 mm, length 260 mm, volume 2.5 l) with medical air constantly flushed through it at a flow rate of 2.5 l/min. The output flow was divided into two lines using a T-connector. One line was attached to a differential pressure amplifier (model 24PCO1SMT, Honeywell Sensing and Control, Golden Valley, MN, USA), while the other line was open to the room air. For semi-quantitative assessment of animals' motor activity, a piezoelectric pulse transducer was placed under the plethysmograph. The transducer was sensitive enough to detect even minor movements (e.g. turning the head), while locomotion produced large oscillatory responses.

### Experimental protocol

Initially, HAB and LAB rats were tested on the elevated plus-maze to confirm their anxiety-related phenotype. The elevated plus-maze, validated for measuring anxiety [20], consisted of 4 elevated arms (100 cm above the floor, 50 cm long and 10 cm wide) arranged in a cross-like position, with two opposite arms being enclosed (by means of 40-cm high walls), and two being open, including at their intersection a central square platform (10×10 cm) which gave access to the four arms. Each rat was initially placed on the central platform facing one closed arm and behaved freely for 5 min. The behavior during the test was recorded using a video camera positioned above the maze. The following behavioral parameters were calculated: i) number of entries in the open arms (% of total entries), ii) latency to enter an open arm (s), and iii) time spent in the open arms (% of total time).

One week after the behavioral testing, rats were placed into the plethysmographic chamber and allowed to explore the new environment for 40 min [19]. Subsequently, the following stimuli were presented: a) a predator (hawk) call was played back for 50 s, and then repeated again 5 min later; b) a piece of cat feces was placed in a syringe, and the air with the cat odor was quickly injected into the input line (through which the plethysmographic chamber was constantly flushed with medical air). All stimuli were separated by at least 5-min intervals and were presented when animals were in a quiet but awake state (i.e., no motor activity, eyes opened, slow regular breathing). After the last stimulus, animals were removed from the plethysmograph and introduced into a restrainer (wire-mesh tube; inner diameter: 6 cm, length: 180 mm), which was immediately placed back into the plethysmograph for 15 minutes. Subsequently, animals were released from the restrainer and allowed additional 15 min in the plethysmographic chamber. All experiments were carried out during the dark phase of the light/dark cycle, with just sufficient levels of red light to permit observation of the animals.

### Data acquisition and analysis

Analogue respiratory and motion signals were digitized at 1 KHz and acquired using a PowerLab A/D converter and ChartPro 6.0 software (ADInstruments, Sydney, Australia). They were low-pass filtered at 20 Hz to remove noise, using a digital filter. Data analysis was performed as follows.

#### a) First 40 min in the plethysmographic chamber

We first calculated the respiratory rate from the respiratory signal. The respiratory rate (cycles per minute, cpm) was measured by calculating the rate of pressure fluctuations inside the chamber. Next, we split the 40-min period into 5-min epochs (0–5 min, 5–10 min, etc.) and constructed histograms (bin width 5 cpm) of respiratory rate vs. time. These histograms show how much time rats spent at a given respiratory rate. Histogram mode peak values were then averaged for each 5-min epoch and plotted against time to assess the time course of the dominant respiratory rate during the first 40 min. For each epoch, we then selected 250 cpm as an approximate centre between low-frequency (0–250 cpm) and high-frequency (251–600 cpm) respiratory rate, the latter reflecting sniffing behavior. This allowed us to calculate the time spent by the animals at high-frequency sniffing mode (expressed as % of total time).

Finally, the motion signal was rectified for each 5-min epoch using IGOR Pro 5.0 software (Wavemetrics, Inc., OR, USA). After setting the threshold level (defined as 150% of the signal when there was no motion), the total duration of time during which the signal exceeded this threshold was determined automatically, and defined as “motion time”. We could not accurately determine the intensity of animals' movements as the amplitude of the motion signal depended on the position of the animal in the plethysmographic chamber.

#### b) Acoustic (predator call) and olfactory (cat odor) stimuli

Mean respiratory rate was calculated from the respiratory signal and expressed as a mean of 5-sec intervals during predator calls (50 s) and cat odor exposure (60 s). In addition, respiratory rate responses to predator calls were analyzed by calculating the area under the response curve (AUC). We could not perform histogram analysis for these data because of the relatively short periods used for assessment.

#### c) Restraint test

The respiratory rate was calculated from the respiratory signal before (5 min) and during (15 min) the restraint test. Next, we split the 15-min restraint period into 5-min epochs (0–5 min, 5–10 min, 10–15 min) and constructed histograms (bin width 5 cpm) of respiratory rate vs. time as described above. In each group, histogram mode peak values were averaged for each 5-min epoch and plotted against time to assess the dominant respiratory rate. Finally, we calculated the time spent by the animals at high-frequency sniffing mode (expressed as % of total time).

#### d) Tidal volume

We also determined relative changes in tidal volume provoked by sensory and stressful stimuli. We were unable to assess the absolute values of tidal volume as this required measurements of body temperature and chamber air humidity [21]. However, we assumed that for short-term recordings, as in the case of predatory calls (50 s), cat odor exposure (60 s) and first minutes of restraint, these variables were constant and thus changes in chamber pressure were only determined by inspiratory and expiratory movements. Tidal volume changes were quantified as % of variation to baseline.

#### e) Sighs

Finally, for each recording period (first 40 min in the plethysmograph, predator calls, cat odor exposure, restraint and post-restraint phases) we quantified the number of sighs (“augmented breaths”). A sigh is a readily identifiable respiratory event: it consists of a deep additional inspiration that starts at or around the peak of a normal respiratory cycle. This superimposition of two inspirations makes a sigh much larger than the preceding and following breaths. A sigh is also usually accompanied by a post-sigh apnea.

All data are presented as mean ± SEM. Statistical significance was set at p<0.05. Two-way ANOVA for repeated measures, with ‘group’ as between-subject factor (two levels: HAB and LAB) and ‘time’ as within-subject factor was applied for respiratory data obtained during: (i) first 40 min in the plethysmographic chamber; (ii) predator calls; (iii) cat odor exposure; (iv) restraint test. After ANOVAs, comparisons between the two groups were conducted using Student's ‘‘t’’-tests, with a Bonferroni correction for multiple comparisons. Student's “t”-tests, following a Levene test, were applied for comparisons between HAB and LAB rats on: (i) data obtained from the elevated plus maze, (ii) number of sighs, (iii) AUC values for respiratory rate responses to predator calls.

## Results

### Behavior on the elevated plus maze

**Figure 1:**
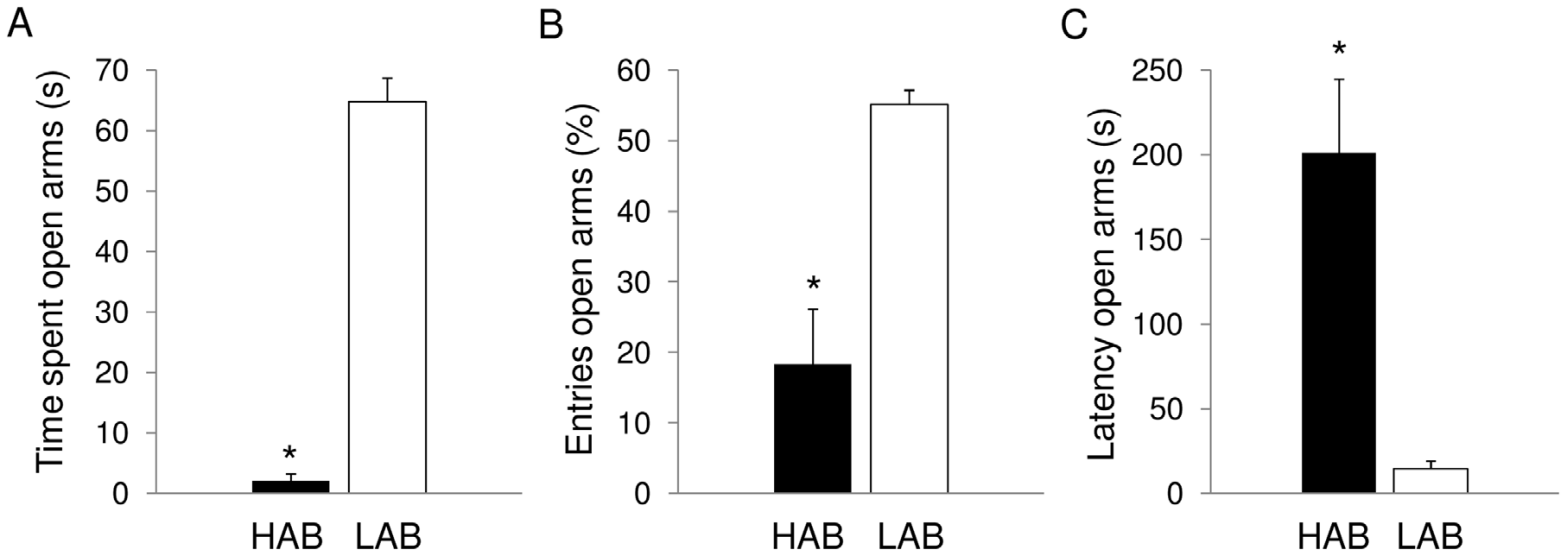
Behavior on the elevated plus maze. For high-anxiety behavior (HAB, n = 10) and low-anxiety behavior (LAB, n = 10) rats, data are expressed as means (±SEM) of: (A) time spent in the open arms (% of total time), (B) number of entries in the open arms (% of total entries), and (C) latency to enter an open arm (s). * indicates a significant difference between HAB and LAB rats (p<0.01).

HABs' and LABs' performance on the elevated plus-maze is illustrated in Fig. 1. This test was conducted as the validation criterion for their relative anxiety phenotype. HAB rats spent less time in the open arms (t = −15.4, p<0.01) and entered them less frequently (t = −4.6, p<0.01) than LABs. In addition, the latency to enter an open arm was longer in HABs compared to LABs (t = 4.2, p<0.01). This clearly indicates that HAB rats were more anxious than LABs, as open/unprotected arms are interpreted as more threatening than the closed/protected arms [20].

### Behavior and respiration during the first 40 min into the plethysmograph

**Figure 2:**
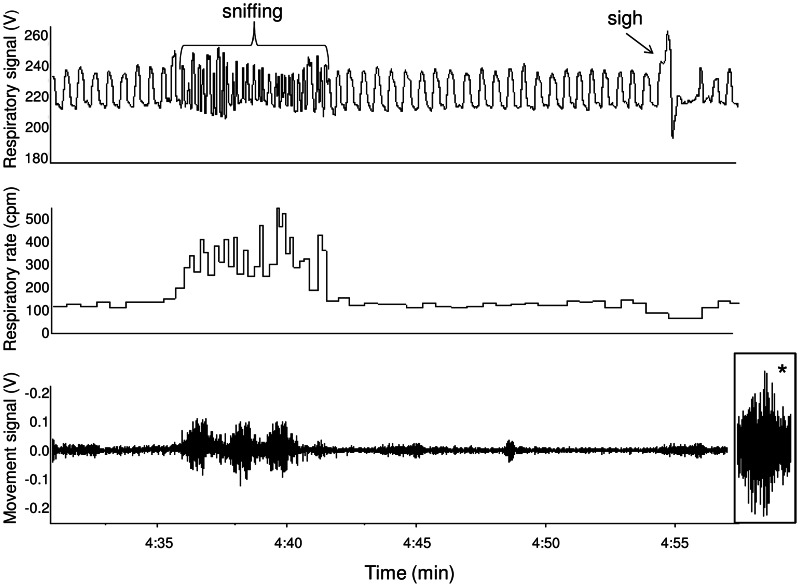
Raw data records of respiratory signal, respiratory rate and motor movements. These data were obtained from a representative high-anxiety behavior rat during free exploration of the plethysmographic chamber. The respiratory signal (top trace) indicates: i) slow regular breathing (referred in the text as “dominant respiratory rate”), ii) sniffing behavior, and iii) a sigh. The movement signal (bottom trace) is from a piezoelectric sensor positioned under the plethysmographic chamber. Note that only small movements occurred during episodes of sniffing (for comparison, the effect of locomotion is shown at the end of the bottom trace (asterisk)).

The behavior of HAB and LAB rats differed over the first 40 minutes inside the plethysmograph. During the first 20 min after entering the chamber (‘active phase’) animals were clearly engaged in exploratory behavior, which was characterized by periods of motor activity or repeated sniffing (head up, frequent movement of vibrissae) intermingled with periods of relative rest (Fig. 2). Of note, sniffing behavior provoked a marked increase in instantaneous respiratory rate (>250 cpm) and coincided with small body movements (Fig. 2). The total duration of motion time during the first 5 min of the active phase was similar between the two groups (HAB = 49±17 s vs. LAB = 59±9 s). During the following 20 minutes animals were more in a state of quiescence (‘resting phase’), sometimes they curled up and closed their eyes, suggesting that they were asleep. The total duration of animals' motion time during the last 5 min of the resting phase was small, with no differences between the two groups (HAB = 4±2 s vs. LAB = 5±2 s).

**Figure 3:**
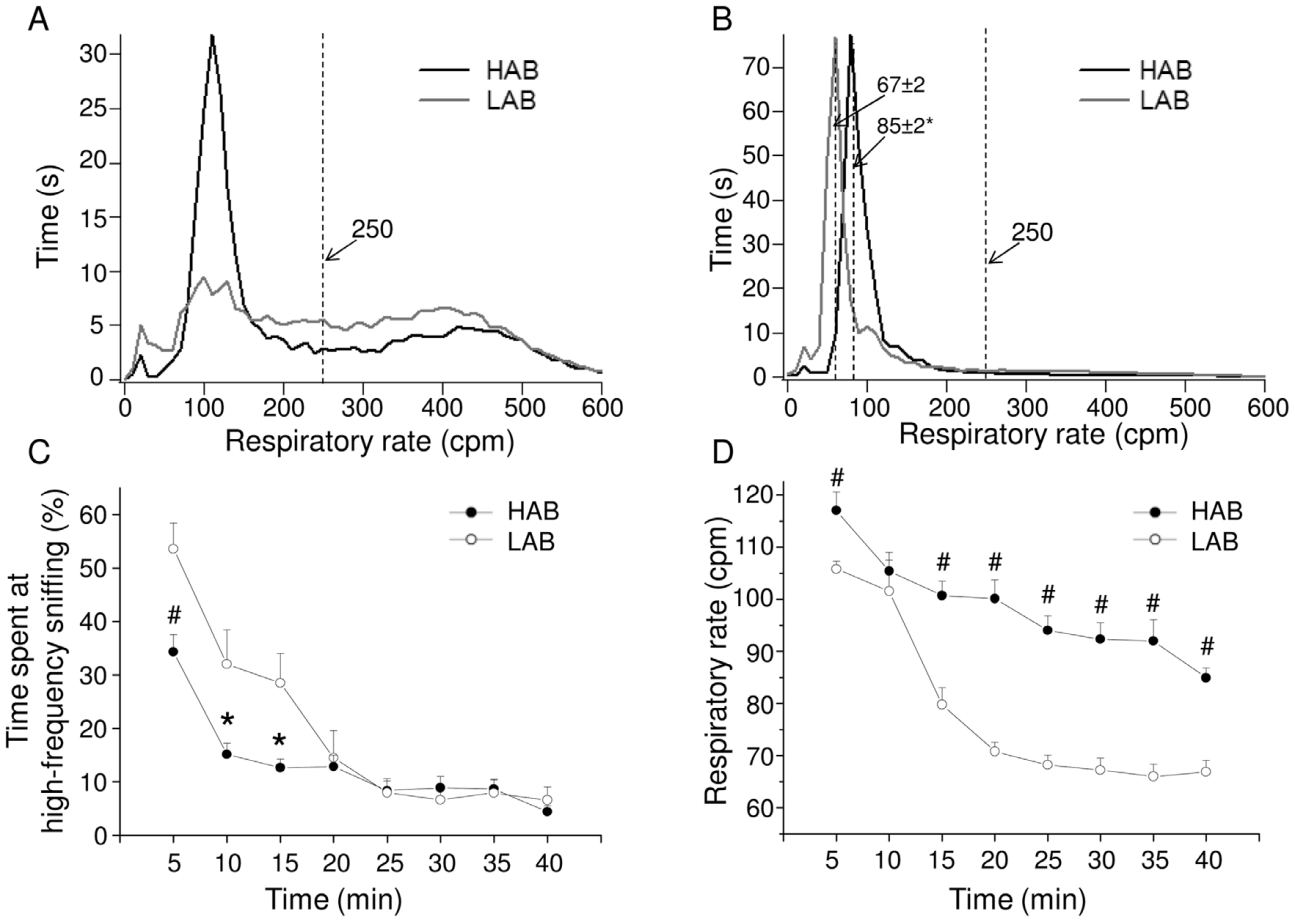
Respiratory patterns during the first 40 min in the plethysmograph. Panels (A) and (B) show how much time high-anxiety behavior (HAB, n = 10) and low-anxiety behavior (LAB, n = 10) rats spent at a given respiratory rate during the first and the last 5 min of free exploration of the new environment, respectively. Panel (C) depicts the time course of changes in the time spent by the animals at high-frequency sniffing, expressed as % of total time (5-min epochs). Panel (D) illustrates the time course of changes in the dominant respiratory rate (i.e. the mode of the low-frequency peaks, similar to those indicated in (B)). Results of ANOVA: (i) significant effect of ‘time’ (F = 24.9, p<0.01) and of ‘group’ (F = 5.7, p<0.05) for values relative to the % of time spent at high-frequency sniffing (Panel C); (ii) significant effect of ‘time’ (F = 106.7, p<0.01), of ‘group’ (F = 76.2, p<0.01) and a ‘time x group’ interaction (F = 4.5, p<0.05) for respiratory rate values (Panel D). ^#^ and * indicate a significant difference (Student ‘t’ test) between HAB and LAB rats (p<0.01 and p<0.05, respectively).

The respiratory patterns of HAB and LAB rats during this 40-min period are illustrated in Fig. 3. During the first 5 min of the ‘active phase’, animals spent time both at low (<250 cpm) and high (>250 cpm) respiratory rate (Fig. 3A), whereas during the resting phase animals spent time almost exclusively at low respiratory rate (Fig. 3B). During the first 15 minutes into the plethysmograph, HAB rats spent less time at high-frequency sniffing mode than LABs (0–5 min: t = −3.2, p<0.01; 5–10 min: t = −2.4, p<0.05; 10–15 min: t = −2.7, p<0.05) (Fig. 3C). Subsequently, no differences between the two groups were found in the amount of time spent at high-frequency sniffing mode (Fig. 3C).

The time course of changes in the dominant respiratory rate (i.e. the mode of the frequency histogram) during the 40 min in the plethysmographic chamber is shown in Fig. 3D. The dominant respiratory rate was higher in HABs than LABs during the first 5 min of the initial testing (t = 2.9, p<0.01). Subsequently, we observed a progressive reduction in the dominant respiratory rate in both groups that was clearly faster in LABs compared to HABs. At the end of the 40-min period, the dominant respiratory rate was significantly higher in HABs than LABs (t = 6.2, p<0.01).

Finally, the very low peak (<40 cpm) on the histograms (Fig. 3A, B) was not an experimental error; it originated from apneic periods that followed augmented breaths (sighs) (Fig. 2). The incidence of sighs during the 40 min in the plethysmographic chamber was significantly larger in HABs than LABs (t = 4.6, p<0.01) (Table 1).

**Table 1:** Incidence of sighs during the experimental procedures.

	New environment	Predator call 1	Predator call 2	Cat odor	Restraint	Post-restraint
HAB	0.49±0.04 ^#^	1.08±0.18^ #^	0.33±0.14	1.2±0.2^ *^	0.66±0.05^ #^	0.50±0.03^ #^
LAB	0.27±0.03	0.17±0.11	0.25±0.13	0.6±0.2	0.41±0.05	0.28±0.04

For high-anxiety behavior (HAB, n = 10) and low-anxiety behavior (LAB, n = 10) rats, data are expressed as means (±SEM) of number of sighs/min. ^#^ and * indicate a significant difference (Student ‘t’ test) between HAB and LAB rats (p<0.01 and p<0.05, respectively).

### Respiratory responses to predator calls

**Figure 4:**
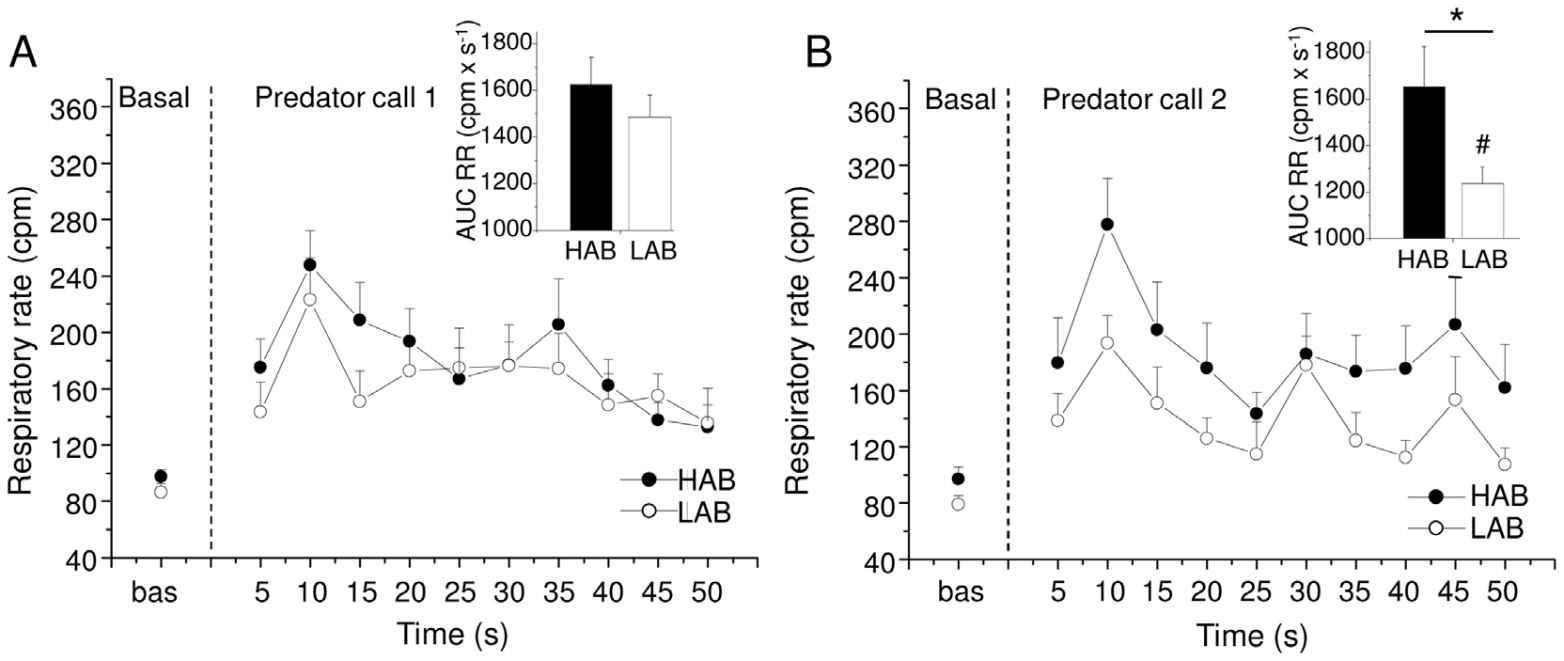
Respiratory rate changes during predator calls. For high-anxiety behavior (HAB, n = 10) and low-anxiety behavior (LAB, n = 10) rats, data are expressed as means (±SEM). Baseline reference value is the mean of the 60 s prior to stimulus onset. During the first (A) and second (B) predator call, each point represents the mean of 5-s intervals. Inner graphs in (A) and (B) represent the area under the response curve (AUC) of respiratory rate during predator calls. Two-way ANOVA yielded a tendency for a group difference in respiratory rate values between HABs and LABs during the second predator call (F = 3.7, p = 0.07). * indicates a significant difference between HAB and LAB rats (Student ‘t’ test, p<0.05). ^#^ indicates a significant difference in AUC values between the first and second predator call in LAB rats (Student ‘t’ test, p<0.05).

During the first predator call HABs and LABs spent the same amount of time at high-frequency sniffing mode (HAB = 16±6% vs. LAB = 13±6% of total stimulus duration). In addition, mean respiratory rate during the first predator call was similar between the two groups (Fig. 4A). However, during the first call all ten HAB rats sighed while only two out of ten LAB rats emitted just one sigh. Consequently, the incidence of sighs was larger in HABs than LABs (t = 4.4, p<0.01) (Table 1).

When the predator call was played back again five minutes later, HABs and LABs spent the same amount of time at high-frequency sniffing mode (HAB = 15±6% vs. LAB = 11±6% of total stimulus duration). However, mean respiratory rate during the second predator call was somewhat higher in HAB rats than LABs (Fig. 4B), with AUC values being significantly higher in HABs than LABs (t = 2.1, p<0.05) (Fig. 4B). Also, in HAB rats AUC values in response to the first and second predator call were similar, whereas in LAB rats AUC values were significantly lower in response to the second predator compared to the first response (t = −2.2, p<0.05) (Fig. 4A, B). During the second predator call, no differences were found both in the number of animals that sighed and in the incidence of sighs between the two groups (Table 1). In addition, we observed in the two groups a similar increase of tidal volume compared to the respective baseline levels during both predator calls (first predator call: HAB = +100±18% vs. LAB = +194±24%; second predator call: HAB = +22±14% vs. LAB = +32±20%).

### Respiratory response to cat odor

**Figure 5:**
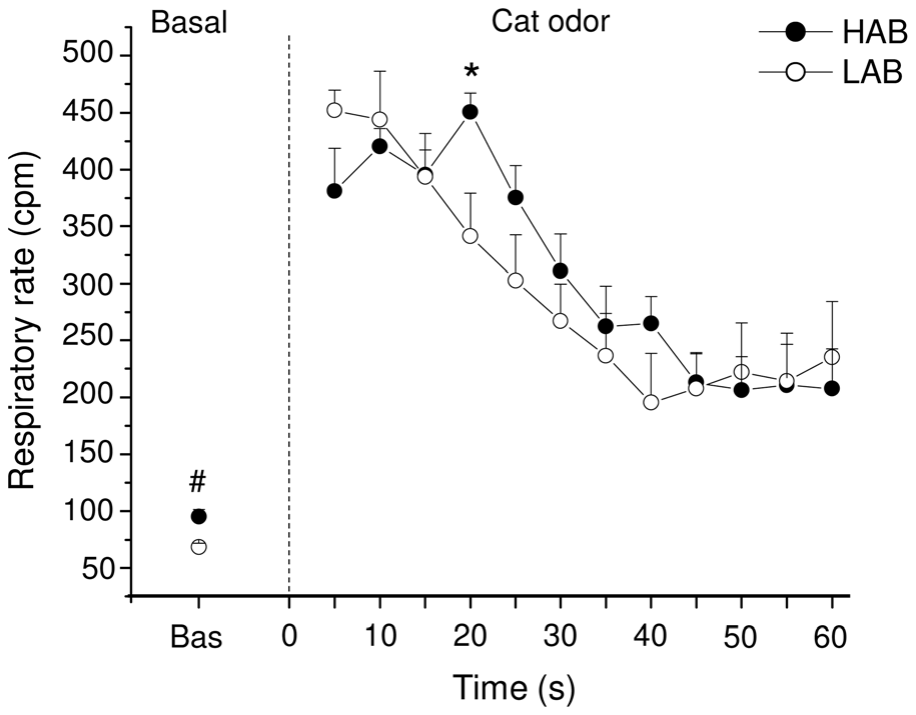
Respiratory rate changes after cat odor exposure. For high-anxiety behavior (HAB, n = 10) and low-anxiety behavior (LAB, n = 10) rats, data are expressed as means (±SEM). Baseline reference value is the mean of the 60 s prior to stimulus onset. During cat odor exposure, each point represents the mean of 5-s intervals. Two-way ANOVA yielded a significant effect of time (F = 24.6, p<0.01). ^#^ and * indicate a significant difference between HABs and LABs (p<0.01 and p<0.05, respectively; Student ‘t’ test).

Prior to stimulus onset HAB rats had significantly higher respiratory rate than LABs (t = 3.6, p<0.01) (Fig. 5). Exposing rats to cat odor evoked a high degree of odor-sampling sniffing, with HAB and LABs rats that spent a similar amount of time at high-frequency sniffing mode (HAB = 66±6% vs. LAB = 64±7% of total stimulus duration). The peak respiratory rate reached during cat odor exposure was similar between the two groups (Fig. 5), with HABs having higher values of respiratory rate than LABs only during the fourth 5-s interval after stimulus onset (t = 2.7, p<0.05) (Fig. 5). In addition, during cat odor exposure we observed in HAB and LAB rats a similar increase of tidal volume compared to the respective baseline levels (HAB = +92±13% vs. LAB = +122±23%).

All HAB rats sighed during cat odor exposure, whereas only five out of nine LAB rats emitted just one sigh. Consequently, the incidence of sighs was significantly larger in HABs than LABs (t = 2.9, p<0.05) (Table 1).

### Respiratory response to the restraint test

**Figure 6:**
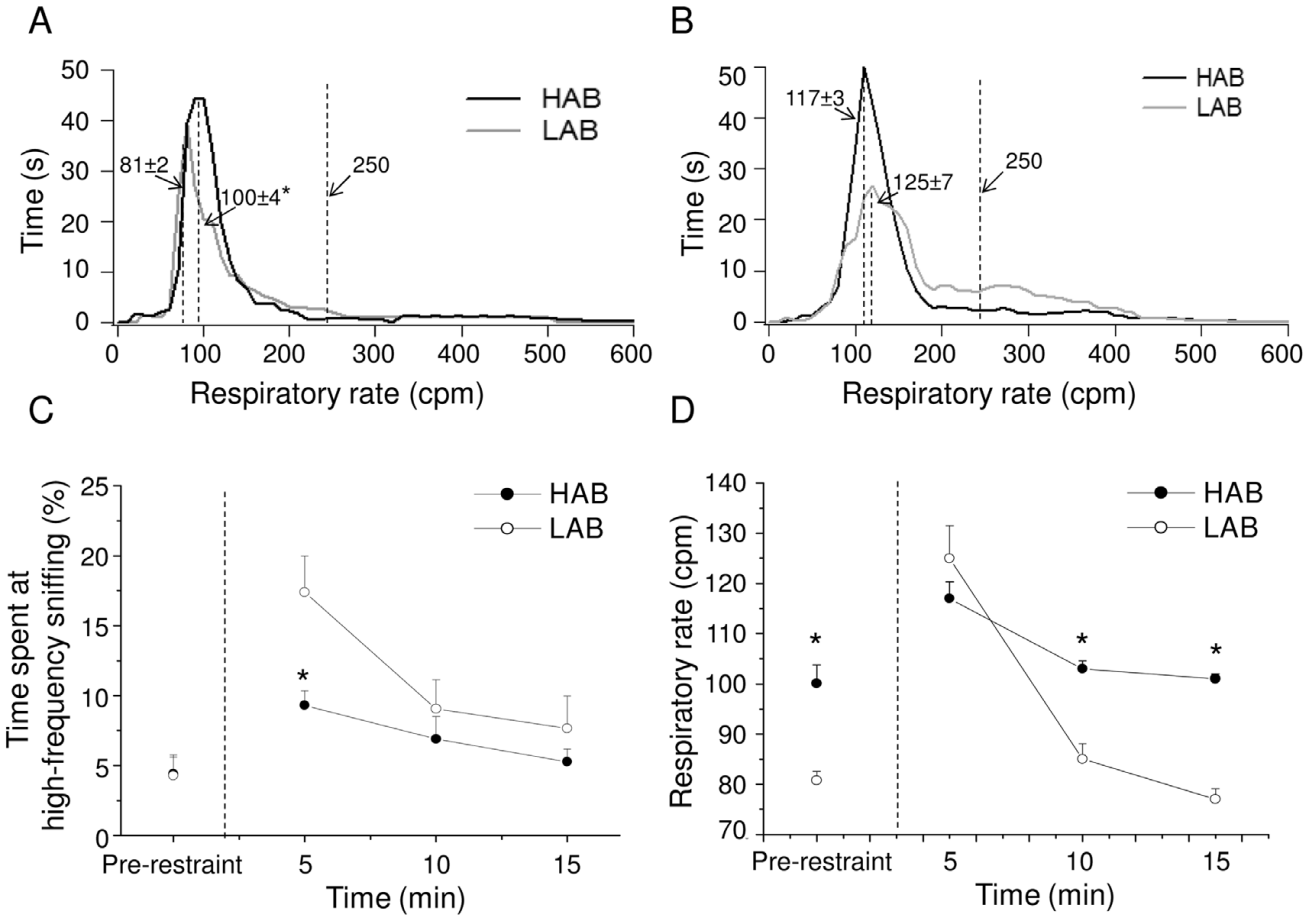
Respiratory patterns before and during the restraint test. Panels (A) and (B) show how much time high-anxiety behavior (HAB, n = 10) and low-anxiety behavior (LAB, n = 10) rats spent at a given respiratory rate during: (A) 5-min pre-restraint recording and (B) first 5-min test recording. Panel (C) depicts the time course of changes in the time spent by the animals at high-frequency sniffing mode before and during the restraint test, expressed as % of total time (5-min epochs). Panel (D) illustrates the time course of changes in the dominant respiratory rate (i.e. the mode of the low-frequency peaks). Results of ANOVA: (i) significant effect of ‘group’ (F = 5.6, p<0.05) for values relative to the % of time spent at high-frequency sniffing (Panel C); (ii) significant effect of ‘time’ (F = 13.2, p<0.01), of ‘group’ (F = 18.8, p<0.01) and a ‘time x group’ interaction (F = 5.4, p<0.05) for respiratory rate values (Panel D). * indicates a significant difference between HAB and LAB rats (Student ‘t’ test, p<0.01).

During the 5 min that preceded the test, animals spent time almost exclusively at low respiratory rate (Fig. 6A). However, HAB rats had significantly higher dominant respiratory rate than LABs (t = 10.2, p<0.01) (Fig. 6A, D). During the first 5 min in the restrainer, HAB rats spent less time at high-frequency sniffing mode compared to LABs (t = −2.95, p<0.01) (Fig. 6C). However, the two groups had similar dominant respiratory rate (i.e. the mode of the low-frequency histogram) during this time (Fig. 6B, D). The magnitude of the stress-induced increase in the dominant respiratory rate compared to pre-restraint values was significantly larger in LABs than HABs (HAB = 17±5 cpm vs. LAB = 44±6 cpm, t = 3.3, p<0.01). During the following 10 min in the restrainer, HABs and LABs spent similar amount of time at high-frequency sniffing mode (Fig. 6C). However, the dominant respiratory rate was significantly higher in HABs than LABs (5–10 min: t = 5.3, p<0.01; 10–15 min: t = 10.2, p<0.01) (Fig. 6D).

Submitting rats to restraint provoked in HAB and LAB rats a similar increase of tidal volume compared to the respective baseline levels (HAB = +39±10% vs. LAB = +48±18%).

All animals emitted sighs during and after the restraint test, with the incidence of sighs being significantly higher in HABs than LABs in both circumstances (t = 3.8, p<0.01; t = 4.6, p<0.01; respectively) (Table 1).

## Discussion

In this study we present a detailed description of the respiratory function in a unique animal model of anxiety, the HAB/LAB rats. Our major novel finding is that HAB and LAB rats differed quite dramatically in terms of the breathing pattern at rest and during arousal and stress. HAB rats had elevated resting respiratory rate, sighed more frequently, showed reduced sniffing in a novel environment and no habituation of the respiratory response to repetitive stimuli compared to LAB rats. These findings support the idea that respiratory indices may represent a promising physiological index of anxiety in rats.

The behavior of HAB/LAB rats on the elevated plus-maze confirms extensive literature documenting clear differences in the level of anxiety between these two rat lines [13], [14]. We found that in HAB/LAB rats respiratory indices show discriminative ability of different anxiogenic situations, thus supporting the idea that this rodent model represents a useful tool for investigating respiratory correlates of anxiety.

In rats – rodents that heavily rely on their olfaction for assessing environment – the respiratory pattern consists of normal (eupnoea) or rapid (tachypnoea) breathing (that we name “dominant” respiratory rate), intermingled with periods of sniffing of variable intensity and duration. The instantaneous respiratory frequency may thus greatly vary – from 60–80 to more than 500 cpm. Consequently, the mean respiratory rate calculated during a given period is strongly affected by the proportion of time spent by an animal at high-frequency sniffing mode. For this reason, in analyzing our data we employed histogram analysis, which allowed us more accurate characterization of the respiratory pattern.

In interpreting our results obtained during the initial period of recording in the plethysmograph, it must be acknowledged that this was not true “resting” or “basal” state as animals were removed from their home cages, placed in a new environment and presumably experienced certain amount of stress. We found that during the first 15 minutes into the plethysmograph HAB rats spent relatively less time at high-frequency sniffing mode than LABs. Previous studies have demonstrated that HAB and LAB rats differ in their coping strategies, with HABs displaying reduced exploratory drive and preferring more passive strategies [14], [22]. Our hypothesis is that the reduction in exploratory sniffing that we observed in HAB rats might thus be a function of a decreased motivational state in these animals and interpreted as a sign of preference for passivity, a behavior that is commonly taken as an indicator of increased anxiety [14]. In addition, HAB rats exhibited higher dominant respiratory rate (i.e., the mode low-frequency peak) than LABs both during the initial testing in the new environment and after animals had settled down (i.e., after about 15 minutes). Clearly, respiratory rate and motor activity are tightly interlinked [19] and it may be speculated that the difference in the dominant respiratory rate observed between the two groups were determined by different somatomotor activity. However, at the end of the 40-min period, when animals were clearly in a resting state and only minor movements could be detected, the dominant respiratory rate was still significantly higher in HABs than in LABs. Thus, physical activity alone cannot be responsible for the difference between HABs and LABs in the dominant respiratory rate, which rather represents a distinctive feature of these animals. Based on our previous observations with cardiovascular responses [19], we consider data values during that period as a reasonable approximation of their true basal respiratory rate.

Interestingly, dominant respiratory rate was higher in HABs than LABs also prior to cat odor exposure and the restraint test, suggesting that the elevated dominant respiratory rate that was found in HAB rats thorough the experimental protocol might also be a consequence of a latent fear induced by the preceding stimuli. This in line with a psychophysiological perspective that the elevated dominant respiratory rate in HAB rats may be part of the increased arousal characteristic commonly observed in anxious individuals, independently from specific emotional contents [23], [24].

During the restraint test we found differences in the respiratory pattern of the two groups that were qualitatively similar to those seen during the initial period in the plethysmograph. Specifically, during the initial confinement of the animals into the restrainer, HAB rats spent relatively less time at high-frequency breathing than LAB rats. In addition, HAB rats exhibited a smaller stress-induced increase in the dominant respiratory rate than LABs. Similar to our argument presented above, we hypothesize that this finding may reflect a difference in the behavioral strategy adopted by the two groups to cope with stress. Several studies have reported, for example, that HAB rats float more and struggle less during a forced swimming test, whereas LAB rats show the opposite and are more active [18], [25], [26]. Our hypothesis is that the reduced respiratory responsiveness seen in HAB rats during the restraint test may be a consequence of their supposed passive (or reactive) style of coping with a stressor.

HAB and LAB rats showed similar respiratory response to the first predator call. However, when the acoustic stimulus was repeated 5 minutes later, we observed a habituation-like effect for the respiratory rate in LAB rats that was not found in HAB rats. This suggests that HAB rats did not adapt in terms of respiratory responsivity to an alerting stimulus, although this was unchanged over time. Persisting, high respiratory reactivity to stress has been described in high-trait anxious individuals [27], [28].

When exposed to cat odor, HAB and LAB rats showed a similar high degree of odor-sampling sniffing. As a consequence of this threat-detection behavioral pattern, the peak respiratory rate reached during this olfactory stimulus was much higher compared to those reached during the acoustic stimuli. Similar bouts of high-frequency respiration in response to novel odorants have been found in previous studies, which have highlighted the importance of sniffing behavior in rats for odor detection and identification [12], [29], [30], [31]. The lack of differences between HAB and LAB rats in the respiratory rate response to the cat odor is likely due to ceiling effect (i.e., the respiratory rate reached its physiological maximum), which may have masked possible subtle differences between the two groups.

Our study is the first to demonstrate that similar to humans, rats do sigh in stressful situations. Furthermore, and also similar to humans, it appears that this sighing occurs more frequently in animals with higher innate anxiety levels. Indeed, a robust and stable difference between HAB and LAB rats was reflected by the incidence of sighs, which was significantly higher in HABs both during the first 40-min recording and under any stressful condition. Sighing is a fundamental vertebrate behavior that can be facilitated by lower blood O_2_ in rats [32] and also by increased blood CO_2_ in other species [33], [34], whose function is to prevent atelectasis in hypoventilated parts of the lungs. Extensive evidence from the human literature has shown that the incidence of respiratory sighing is greater among anxious patients, especially those with diagnoses of panic disorders. Panic subjects sigh more often during resting state [35], [36], [37] and during/after challenges [2] than controls. It has been proposed that a hypersensitive suffocation alarm system may explain the high incidence of sighs in panic disorder [38]. According to this theory, panic disorder subjects might have an overly sensitive chemoreceptor activity and thus may be inclined to take periodic deep breaths to lower the pCO_2_ safely below the threshold level. Evidence that panic disorders subjects might have an overly sensitive chemoreceptor activity comes from clinical studies demonstrating that lower subthreshold concentrations of hypercarbic gas (e.g. 5–7% CO_2_) provoke panic attacks in the majority of panic disorder patients, but not in healthy controls [39], [40], [41]. Other studies have failed to provide unequivocal evidence of a specific, dysregulated suffocation alarm system in panic and have hypothesized that frequent sighing may be a compensatory response in an attempt to reduce the sensation of dyspnea [3], [42]. Our data does not clarify whether increased sighing in HAB rats acts as a general re-setter of the respirator system and can be explained by respiratory variables, such us hypoxia or hypercapnia. On the other hand, our results clearly indicate that rats must possess a mechanism linking perception of stress to the ponto-medullary respiratory pattern generator. We have recently demonstrated in anesthetized rats that pharmacological activation of the dorsomedial hypothalamus (DMH, a crucial “defense area” that coordinates stress-induced autonomic neural responses) results in dramatic increase in the number of sighs as well as in tachypnoea [43]. We thus hypothesize that the stress-evoked respiratory responses that we describe here were in fact triggered by the DMH. A previous study has proposed that sighs are rats' expression of relief and may function as a signal of safety [44]. In our rats, however, sighing was more frequent during stressful conditions (i.e., first minutes in the plethysmograph or restraint) than during periods of relative reduced perception of danger (i.e., end of the 40-min period or post-restraint phase), thus not supporting the relief signal hypothesis of sighing. Like humans, sighing in rats could be due to various causes. Whatever mechanisms predominate, frequent sighing is a respiratory behavior that markedly differentiates between HAB and LAB animals, and resembles what has been observed in panic disorder subjects.

### Conclusion and perspectives

The results of this study complement and extend previous animal findings [7]–[12] documenting that the respiratory phenotype can differ considerably between subjects and that such variability can be due to the individual levels of anxiety-related behavior. It must be acknowledged that the interpretation of the data in this study is limited by the lack of quantitative assessment of total somatomotor activity, which may have partially accounted for the differences observed in the respiratory patterns between HAB and LAB rats. Another limitation of this study is that we have not determined whether the respiratory changes in HAB rats can be attenuated with anxiolytic compounds. On this regard, future work is required in order to validate and strengthen the use of respiration as a reliable, locomotor-independent index of anxiety in rats. Nevertheless, the respiratory changes found in high-anxiety behavior rats share similarities to the symptoms observed in patients with anxiety and panic disorders and provide evidence that respiration may represent a promising method for assessing anxiety states in preclinical studies.
